# LEVERAGING ELECTRONIC MEDICAL RECORD DATA TO IDENTIFY MODIFIABLE CONTRIBUTORS TO ACUTE CARE USE IN DEMENTIA

**DOI:** 10.1093/geroni/igae098.0661

**Published:** 2024-12-31

**Authors:** Stephanie Nothelle, Mohamed Ahmidouch, Julia Loosen, Cynthia Boyd, Jennifer Wolff

**Affiliations:** Johns Hopkins University School of Medicine, Baltimore, Maryland, United States; University of North Carolina School of Medicine, Chapel Hill, North Carolina, United States; Johns Hopkins Bayview Medical Center, Baltimore, Maryland, United States; Johns Hopkins University, Baltimore, Maryland, United States; Johns Hopkins University, Baltimore, Maryland, United States

## Abstract

Persons living with dementia (PLWD) are at increased risk of potentially preventable acute care visits (hospitalizations and emergency department visits), but modifiable contributors are poorly understood. We aimed to identify potentially modifiable factors from patient secure portal messages and outpatient clinical notes in the 30 days leading up to a potentially preventable acute care visit. We examined data from the Johns Hopkins Health System electronic medical record from 2017-2022. We included adults >64 years with dementia diagnosis, 1+ advanced chronic illness (advanced liver disease, chronic obstructive pulmonary disease, congestive heart failure, diabetes with complications, chronic kidney disease), 2+ primary care visits who had a potentially preventable acute care visit. Two coders coded each message or note using a qualitative content analysis approach. We included 128 PLWD of whom 75 (69%) were female, and 68 (53%) were white. Approximately 47 (37%) had at least one patient/caregiver initiated portal message. Topics identified were similar across patient portal message and clinical notes. The most commonly mentioned needs related to care coordination, health systems navigation, medication management, access to and timeliness of care, and new physical symptoms. Cognitive concerns or symptoms were more commonly mentioned in clinical notes than portal messages. Portal messages and outpatient clinical notes prior to a potentially preventable acute care visit contain information about unmet care needs, predominantly related to coordinating and accessing care. Needs identified in this analysis may be addressable by a care manager to prevent unnecessary acute care use.

